# The COVID-19 lab score: an accurate dynamic tool to predict in-hospital outcomes in COVID-19 patients

**DOI:** 10.1038/s41598-021-88679-6

**Published:** 2021-04-30

**Authors:** Pablo Jose Antunez Muiños, Diego López Otero, Ignacio J. Amat-Santos, Javier López País, Alvaro Aparisi, Carla E. Cacho Antonio, Pablo Catalá, Teba González Ferrero, Gonzalo Cabezón, Oscar Otero García, José Francisco Gil, Marta Pérez Poza, Jordi Candela, Gino Rojas, Víctor Jiménez Ramos, Carlos Veras, J. Alberto San Román, José R. González-Juanatey

**Affiliations:** 1 Cardiology Department, Hospital Universitario de Santiago de Compostela, Choupana S/N, C.P. 15706, A Coruña, Spain; 2 Cardiology Department, Hospital Clínico Universitario de Valladolid, Valladolid, Spain; 3 CIBERCV, Madrid, Spain

**Keywords:** Infectious diseases, Medical research

## Abstract

Deterioration is sometimes unexpected in SARS-CoV2 infection. The aim of our study is to establish laboratory predictors of mortality in COVID-19 disease which can help to identify high risk patients. All patients admitted to hospital due to Covid-19 disease were included. Laboratory biomarkers that contributed with significant predictive value for predicting mortality to the clinical model were included. Cut-off points were established, and finally a risk score was built. 893 patients were included. Median age was 68.2 ± 15.2 years. 87(9.7%) were admitted to Intensive Care Unit (ICU) and 72(8.1%) needed mechanical ventilation support. 171(19.1%) patients died. A Covid-19 Lab score ranging from 0 to 30 points was calculated on the basis of a multivariate logistic regression model in order to predict mortality with a weighted score that included haemoglobin, erythrocytes, leukocytes, neutrophils, lymphocytes, creatinine, C-reactive protein, interleukin-6, procalcitonin, lactate dehydrogenase (LDH), and D-dimer. Three groups were established. Low mortality risk group under 12 points, 12 to 18 were included as moderate risk, and high risk group were those with 19 or more points. Low risk group as reference, moderate and high patients showed mortality OR 4.75(CI95% 2.60–8.68) and 23.86(CI 95% 13.61–41.84), respectively. C-statistic was 0–85(0.82–0.88) and Hosmer–Lemeshow p-value 0.63. Covid-19 Lab score can very easily predict mortality in patients at any moment during admission secondary to SARS-CoV2 infection. It is a simple and dynamic score, and it can be very easily replicated. It could help physicians to identify high risk patients to foresee clinical deterioration.

## Introduction

In December 2019, an outbreak of a new syndrome emerged in the Chinese province of Hubei. A new betacoronavirus was described, called SARS-CoV-2^1^. By April 18th, the World Health Organization reported more than 2 million of confirmed cases of COVID-19 disease (Coronavirus Disease 2019), affecting 200 different countries all around the world^2^. Deaths rose to almost 140 thousand people.

One of the most encouraging characteristic of Covid-19 disease, is the huge differences in the clinical manifestations depending of each patient^3–5^. It is suspected that some of them are paucisymptomatic, or even completely asymptomatic. On the other hand, in some patients SARS-CoV-2 could trigger a big inflammation response, causing a severe disease. Severe Acute Respiratory Syndrome is one of the most common and severe complications, due to its predisposition for the respiratory system, requiring sometimes admission in Intensive Care Units (ICU) or mechanical ventilation and with an elevated death rate^6^. Three different phases are described^7^. First one is the early infection, and secondary there is the pulmonary involvement, causing bilateral infiltrates. Finally, a stage of hyperinflammation is described, affecting extrapulmonary organs and triggering a systemic cytokine storm. Elevated levels of some biomarkers^5^ such as Interleukin (IL)-2, -6, -7, TNFα, IFNγ, C-reactive protein, ferritin, D-Dimer, procalcitonin, troponin are described in this severe patients. At this stage, mechanical ventilation and admission at ICU are frequently needed, because of hypoxemia, respiratory failure, or even shock secondary to vasoplegia.

The aim of the study was to identify high risk patients affected with COVID-19, those with a poor outcome, depending on laboratory biomarkers. Mortality was selected as the primary endpoint of interest. Risk factors and comorbidities such as obesity and diabetes have been described with a poorer outcome^8,9^. Moreover, a dynamic, objective and very easily score could help physicians to anticipate clinical deterioration. Our goal is to stratify the risk of death for patients with Covid-19 at any moment during the admission, using a very simple and reproducible analytic tool, developed only with laboratory parameters.

## Methods

This is a multicentre, retrospective, observational study performed at three university hospitals from the North-West region of Spain, covering a population of more than one million of inhabitants. In our registry we included all the confirmed cases for SARS-CoV-2 infection from our health areas (n = 1779). Patients were recorded during the first two months since the first case in our areas was diagnosed. For the purpose of this study, we only analysed those patients admitted to hospital with available information about laboratory data (n = 893). Follow-up continued for 3 months. This study was approved by the Ethical Committee of Investigation of Santiago and Lugo (registry code 2020/187) with an approval number UNH-ARA-2020-01, and it fulfils the Declaration of Helsinki of 1975.

We selected laboratory parameters were previously associated with SARS-CoV2 infection in recent literature^5,10–14^ and with a poorer outcome similar viral severe infections^15–18^. Moreover, haemoglobin and erythrocyte levels, or serum creatinin were evaluated because of their relation with outcomes in severe ill patients, The most deviant from normal results of the laboratory tests for each biomarker during the admission were selected for the score (they were mostly the higher ones, except for the haemoglobin and erythrocytes).

Normal ranges for the different parameters were: procalcitonin < 0.05 ng/mL; Haemoglobin 13.5–17.5 g/dL for men and 12.2–16.1 g/dL for women; erythrocytes 4.5–5.5 per 10^6^/mm^3^ ; leukocytes 4.09–10.8 per 10^3^/mm^3^ ; neutrophils 1.7–7.33 per10^3^/mm^3^ ; creatinine 0.4–1.3 mg/dL for men and 0.4–1.1 mg/dL for women; C-reactive protein 0–0.5 mg/L; interleukin-6 0–5.0 pg/mL; lactate dehydrogenase (LDH) 200–446 UI/L; D-dimer 0–500 ng/mL.

### Outcomes

This work is conducted to develop an easily replicable, objective and dynamic score to help physicians to assess the risk of adverse outcomes at any moment during the admission due to COVID-19.

All the information about the clinical evolution and the complications developed was recollected along the admission and continued also when discharged, thanks to remote monitoring. Mortality for any cause during the hospitalization was selected as the main outcome, and was recollected from clinical records. Secondary endpoints were non-cardiovascular outcomes such as ICU admission, necessity of mechanical ventilation or pulmonary embolism. Acute myocardial infarct, heart failure and stroke were established as cardiovascular outcomes.

### Data collection

Standardized forms were used for the setting-up of the database, including demographic information, epidemiological data, previous comorbidities and chronic treatments, the clinical data available at the moment when they were admitted to hospital (symptoms, fever and peripheral O2 saturation (SpO2), the results of all laboratory tests done during the admission and the treatment received. All these information was collected from medical records. Laboratory test and clinical decisions were taking according to physician´s criteria. Informed consent was obtained for study participation of each patient when admitted to hospital. The data in source documents was confirmed independently by at least two physicians.

### Statistical analysis

Continuous variables are presented as mean (SD), whereas discrete variables are presented as percentages. Comparisons between discrete variables were performed using the χ2 test or Fisher exact test as required, and comparisons between continuous variables using the Student t-test (Table 1).

**Table 1:** Baseline characteristics of total population (n = 893).

Baseline characteristic	Value
Age	68.2 ± 15.2
Female sex	453 (50.7%)
Obesity	110 (12.3%)
Institutionalized person	15 (1.7%)
Dementia	17 (1.9%)
Dependency	43 (4.8%)
Health worker	13 (1.5%)
Active smoking	40 (4.5%)
Hypertension	444 (49.7%)
Diabetes mellitus	174 (19.5%)
Dyslipidemia	362 (40.5%)
Peripheral artery disease	21 (2.4%)
Heart disease	149 (16.7%)
Ischemic heart disease	75 (8.4%)
Myocardiopathy or depressed LVEF	49 (5.5%)
Valvular heart disease	12 (1.3%)
Atrial fibrillation	20 (2.2%)
Pulmonary disease	55 (6.2%)
COPD or asthma	84 (9.4%)
Prior stroke	19 (2.1%)
Prior cancer	12 (1.3%)
Hypothyroidism	13 (1.5%)
Autoimmune disease	18 (2.0%)
Anticoagulation	201 (11.4%)
Antiplatelet therapy	144 (16.1%)
ACEI/ARB	333 (37.3%)
Antialdosteronic drug	21 (2.4%)
B-blockers	158 (17.7%)
Calcium channel blocker	65 (7.3%)
Diuretic drugs	113 (12.7%)
Statin	294 (32.9%)
Corticosteroid	24 (2.7%)
Inmunosupression	12 (1.3%)
Days of symptoms	7.3 ± 5.1
Fever	562 (62.9%)
Respiratory insufficiency	350 (39.2%)
Antiviral	751 (84.1%)
Chloroquine	772 (86.5%)
Interferon	33 (3.7%)
Tocilizumab	91 (10.2%)
Azithromycin	619 (69.3%))
Ceftriaxone	450 (50.4%)
Corticosteroids	223 (25.0%)
Anticoagulation	347 (38.9%)
Antiplatelets	542 (60.7%)

Categorical variables in n (%) and quantitative variables in mean ± standard deviation.

*LVEF* left ventricle ejection fraction.

Backward stepwise logistic regression analysis was performed to determine the predictive factors for mortality. Variables that were significantly associated with mortality in the univariate analysis (Supplementary Table 1) were included in the multivariate model (Table 2). The multivariate adjustment was performed according these variables, in addition to biomarkers: age, comorbidities (hypertension, dyslipidemia, diabetes mellitus, peripheral artery disease, heart disease, Chronic Obstructive Pulmonary Disease (COPD)/asthma), days of symptoms, respiratory insufficiency (defined as PaO2 < 60 mmHg), and in-hospital drugs (antiviral, chloroquine, ceftriaxone, corticosteroids, anticoagulation, antiplatelet). The incremental value of each significant biomarker, when it was added to a clinical base model, was assessed with the change in the c-index (Table 3).

**Table 2:** Univariate and multivariate analysis for biomarkers to predict mortality.

Laboratory data	Univariate			Multivariate		
	OR	95% CI	p	OR	95% CI	p
Hemoglobin, per g/dL	0.74	0.68–0.80	< 0.001	0.82	0.75–0.91	< 0.001
Leukocytes, per 10^3^/mm^3^	1.11	1.08–1.14	< 0.001	1.08	1.05–1.11	< 0.001
Neutrophils, per 10^3^/mm^3^	1.10	1.06–1.14	< 0.001	1.07	1.03–1.12	0.001
Lymphocytes, per 10^3^/mm^3^	0.31	0.21–0.48	< 0.001	0.72	0.50–1.04	0.083
Platelets, per 10^5^/mm^3^	0.80	0.68–0.95	0.010	1.00	0.99–1.01	0.903
Neutrophil–lymphocyte ratio	1.04	1.02–1.05	< 0.001	1.02	1.01–1.03	0.012
Lymphocytes per 100 leukocytes	0.88	0.86–0.91	< 0.001	0.93	0.90–0.96	< 0.001
Platelet–lymphocyte ratio	1.84	1.19–2.86	0.006	1.14	0.63–2.08	0.665
GOT, per 10 UI/L	1.05	1.02–1.08	< 0.001	1.05	1.01–1.08	0.009
GGT, per 10 UI/L	1.01	1.00–1.02	0.005	1.02	1.00–1.03	0.019
Creatinine, per mg/dL	2.79	2.19–3.57	< 0.001	1.97	1.58–2.47	< 0.001
CRP, per 10 mg/L	1.10	1.08–1.12	< 0.001	1.09	1.06–1.12	< 0.001
IL-6, per 10 pg/mL	1.01	1.00–1.02	0.017	1.01	1.00–1.02	0.014
Ferritin, per 1000 ug/mL	1.25	1.09–1.44	0.002	1.19	0.98–1.43	0.079
Procalcitonin, per ng/mL	1.05	1.02–1.08	0.002	1.05	1.02–1.08	0.001
LDH, per 100 UI/L	1.19	1.12–1.26	< 0.001	1.34	1.21–1.49	< 0.001
D-Dimer, per 1000 ng/mL	1.05	1.03–1.07	< 0.001	1.03	1.01–1.05	0.001

Multivariate adjustment: Age, comorbidities (hypertension, dyslipemia, diabetes mellitus, peripheral artery disease, heart disease, COPD/asthma), days of symptoms, respiratory insufficiency, in-hospital drugs (antiviral, chloroquine, ceftriaxone, corticosteroids, anticoagulation, antiplatelet).

**Table 3:** Model performance to predict mortality basing on the addition of biomarkers.

Model	C-statistic	95% CI	P-value
Base model (as reference)	0.891	0.866–0.916	ref
Base model + hemoglobin	0.900	0.877–0.923	0.012
Base model + leukocytes	0.907	0.884–0.929	0.001
Base model + neutrophils	0.934	0.914–0.955	< 0.001
Base model + neu-lymph ratio	0.934	0.914–0.955	< 0.001
Base model + lymph × 100 leu	0.905	0.884–0.927	0.015
Base model + GOT	0.915	0.892–0.938	0.002
Base model + GGT	0.906	0.881–0.931	0.014
Base model + creatinine	0.910	0.887–0.933	< 0.001
Base model + CRP	0.915	0.892–0.937	0.001
Base model + IL-6	0.916	0.883–0.949	0.021
Base model + procalcitonin	0.916	0.877–0.926	0.026
Base model + LDH	0.925	0.905–0.946	< 0.001
Base model + D-dimer	0.903	0.879–0.926	0.036

Model performance after the addition of different biomarker to the base model [age, comorbidities (hypertension, dyslipidemia, diabetes mellitus, peripheral artery disease, heart disease, COPD/asthma), days of symptoms, respiratory insufficiency, in-hospital drugs (antiviral, chloroquine, ceftriaxone, corticosteroids, anticoagulation, antiplatelet)].

**Figure 1:**
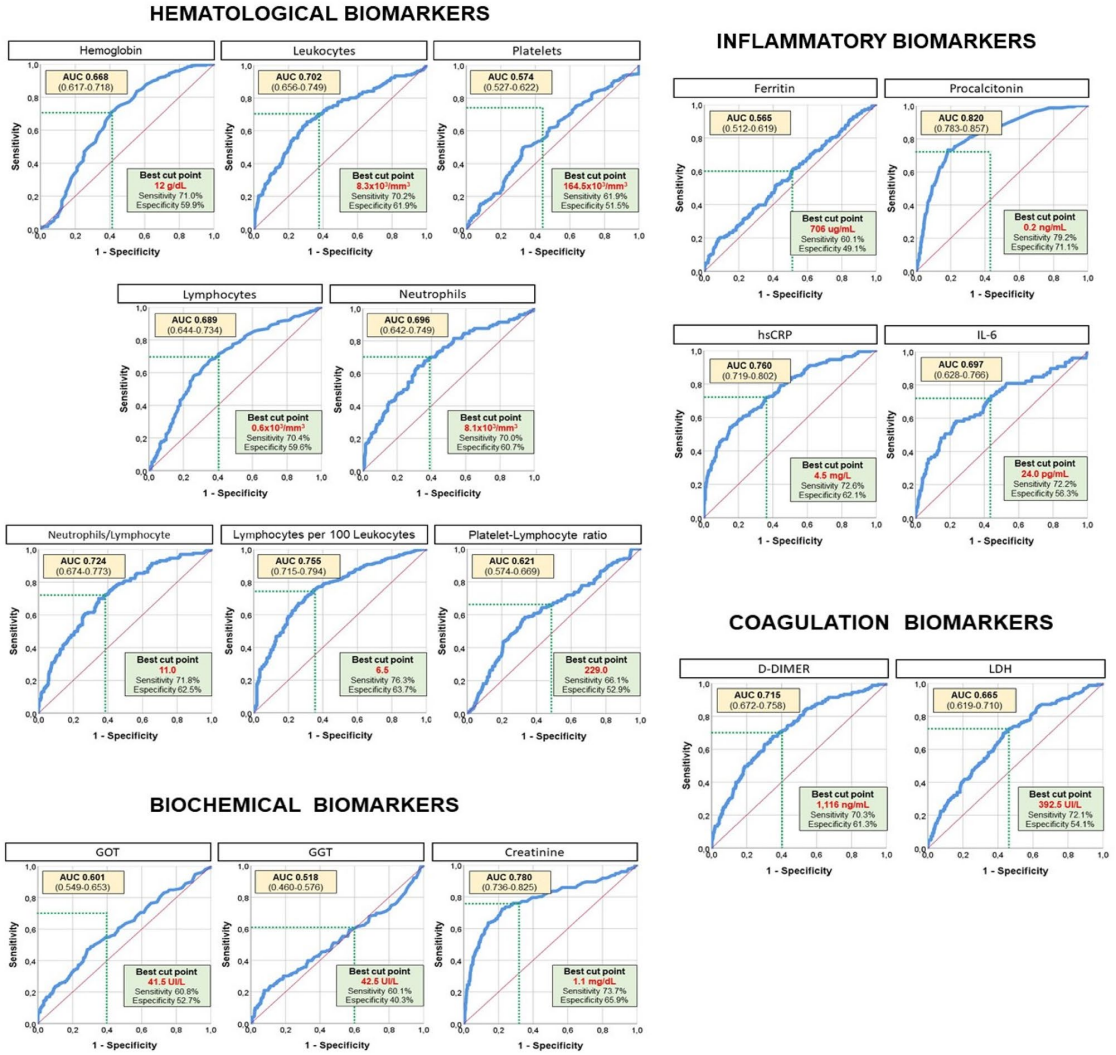
Discrimination of biomarkers.* AUC* area under the curve for each different biomarker.

The optimal cut-point definition for different biomarkers was defined based on the receiver operating characteristic (ROC) curve, using the Youden index (Fig. 1). Sensitivity and specificity were reported for cut-points.

**Figure 2:**
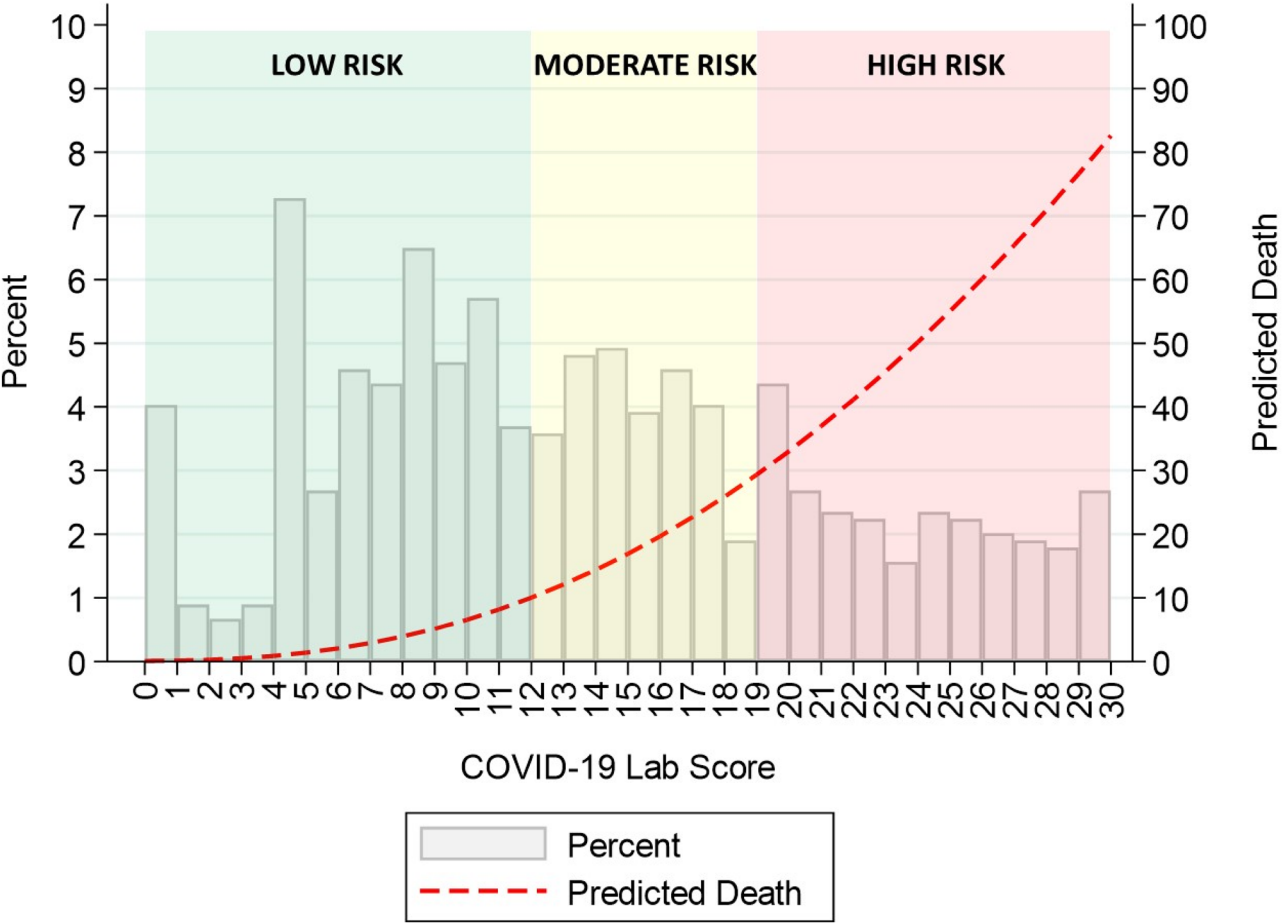
COVID-19 lab score: histogram and predicted mortality. Represents the risk of mortality depending on the score, divided in three different groups.

After categorizing the biomarkers according to their cut-points, those resulting as independent predictors of mortality by the multivariate logistic regression analysis were incorporated into a risk score (Table 4). The scores assigned to each biomarker were determined according to the value of the odds ratio (OR). The predicted probability of death based on the risk score was graphically represented after modelling by fractional polynomials (Fig. 2). The performance of this risk score was tested by assessing its discrimination and calibration capacity (Table 5), for all-cause death. Discrimination was evaluated by calculating the C statistic, and calibration was assessed by the Hosmer–Lemeshow test.

**Table 4:** Multivariate analysis for biomarkers to predict mortality basing on cut-off points (as categorial variables).

Laboratory data	Multivariate			Points
	OR	95% CI	p-value	
Hemoglobin < 12 g/dL	1.07	1.05–1.09	< 0.001	1
Erythrocytes < 4.1 per 10^6^/mm^3^	2.14	1.19–3.84	0.011	2
Leukocytes > 8.3 per 10^3^/mm^3^	2.51	1.56–4.03	< 0.001	3
Neutrophils > 8.1 per 10^3^/mm^3^	2.13	1.14–3.95	0.017	2
Lymphocytes < 6.5 per 100 leukocytes	2.85	1.82–4.46	< 0.001	3
Creatinine > 1.1 mg/dL	4.10	2.56–6.55	< 0.001	4
CRP > 4.5 mg/L	4.05	1.08–8.58	0.035	4
IL-6 > 24 pg/mL	1.83	1.17–2.88	0.009	2
Procalcitonin > 0.2 ng/mL	5.72	3.35–9.76	< 0.001	5
LDH ≥ 393 100 UI/L	4.29	2.49–7.39	< 0.001	4
D-Dimer > 1116 ng/mL	1.92	1.22–3.02	0.005	2

Multivariate adjustment: age, comorbidities (hypertension, dyslipemia, diabetes mellitus, peripheral artery disease, heart disease, COPD/asthma), days of symptoms, respiratory insufficiency, in-hospital drugs (antiviral, chloroquine, ceftriaxone, corticosteroids, anticoagulation, antiplatelet).

**Table 5:** Predictive ability of COVID-19 lab score for mortality.

COVID-19 lab score			Mortality
Odds ratio	Continuous		1.23 (1.19–1.26)
	Categorical	Low risk	Ref
		Moderate risk	4.75 (2.60–8.68)
		High risk	23.86 (13.61–41.84)
Discrimination	C-statistics		0.85 (0.82–0.88)
Calibration	Hosmer–Lemeshow p-value		0.63
	Chi^2^		6.11

All p values were two-sided and values < 0.05 were considered as significant. All statistical analyses were performed using STATA software, version 15.1 (Stata Corp, College Station, Texas, USA), and IBM SPSS software, version 24.0 (IBM, Armonk, New York, USA).

## Results

From all cases diagnosed in our health areas (n: 1799), a total of 893 patients were admitted to hospital due to COVID-19. Baseline characteristics are described in Table 1. Median age was 68.2 ± 15.2 years, and 453 were female (50.7%). Considering these 893 patients admitted to hospital, 87 (9.7%) patients were admitted to Intensive Care Unit (ICU), 72 cases (8.1%) needed mechanical ventilation support and 171 (19.1%) died. Mean length of hospital stay was 10.6 days, but few patients were not discharged at the end of the follow-up.

In relation with cardiovascular outcomes, heart failure was the most common complication (61cases, 6.8%). Acute myocardial infarction, myocarditis and pericarditis were also described (3 (0.3%); 2 (0.2%); 3 (0.3%), respectively). Pulmonary embolism was diagnosed in 8 patients (0.9%).

Univariate analysis with clinical characteristics and treatment to predict mortality was made (Supplementary Table 1). Those that were found to have significant difference (p < 0.05) (age, comorbidities (hypertension, dyslipemia, diabetes mellitus, peripheral artery disease, heart disease, COPD/asthma), days of symptoms, respiratory insufficiency, in-hospital drugs (antiviral, chloroquine, ceftriaxone, corticosteroids, anticoagulation, antiplatelet)) were included in the multivariate analysis.

After multivariate adjustment, most laboratory parameters were significantly associated with mortality (haemoglobin, leukocytes, neutrophils, neutrophils/lymphocytes ratio, lymphocytesx100leucocyte, GOT, GGT, creatinine, CRP, IL-6, procalcitonin, LDH and D-dimer), only lymphocytes, platelets and ferritin values were not associated with high mortality (Table 2). The incremental value of each of these significant biomarkers, when it was added to a clinical base model, was assessed with the change in the c-index, achieving all of them significant difference regarding the reference (Table 3).

In order to convert these variables to categorical ones, the optimal cut-off point definition for each different biomarker was established based on ROC curves, as is represented in Fig. 1.

After categorizing the biomarkers according to their cut-off points, those resulting as independent predictors of mortality by the multivariate logistic regression analysis were incorporated into a risk score (Table 4). The scores assigned to each biomarker were determined according to the value of the odds ratio (OR). For OR between 1.00 and 1.50, 1 point was assigned. For OR between 1.76 and 2.50, 2 points were assigned. For OR between 2.51 and 3.50, 3 points were assigned. For OR between 3.51 and 4.50, 4 points were assigned. And for OR > 4.50, 5 points were assigned.The one with an individual higher score was procalcitonin < 0.2 ng/mL with 5 points (OR 5.72, CI 95% 3.35–9.76, p < 0.001), in relation with the severity of bacterial coinfection. Hemoglogin < 12 g/dL (OR 1.07, CI 95% 1.05–1.09, p < 0.001; 1 point), erythrocytes < 4.1 per 10^6^/mm^3^ (OR 2.14, CI 95% 1.19–3.84, p 0.011;2 points), leukocytes > 8.3 per 10^3^/mm^3^ (OR 2.51, CI 95% 1.56–4.03, p < 0.001; 3 points), neutrophils > 8.1 per10^3^/mm^3^ (OR 2.13, CI 95% 1.14–3.95, p 0.017; 2 points), lymphocytes < 6.5 per 100 leukocytes (OR 2.85, CI 95% 1.82–4.46, p < 0.001; 3 points), creatinine (OR 4.10, CI 95% 2.56–6.55, p < 0.001; 4 points), C-reactive protein > 4.5 mg/L (OR 3.05, CI 95% 1.08–8.58, p 0.035; 4 points), interleukin-6 > 24 pg/mL (OR 1.83, CI 95% 1.17–2.88, p 0.009; 2 points), lactate dehydrogenase (LDH) ≥ 393 UI/L (OR 4.29, CI 95% 2.49–7.39, p < 0.001; 4 points), and D-dimer > 1116 ng/mL (OR 1.92, CI 95% 1.22–3.02, 2 points).

With these individual scores, the COVID-19 Lab score is performed, ranging from 0 to 30 points. The predicted probability of death based on the risk score was graphically represented after modelling by fractional polynomials (Fig. 2).

**Figure 3:**
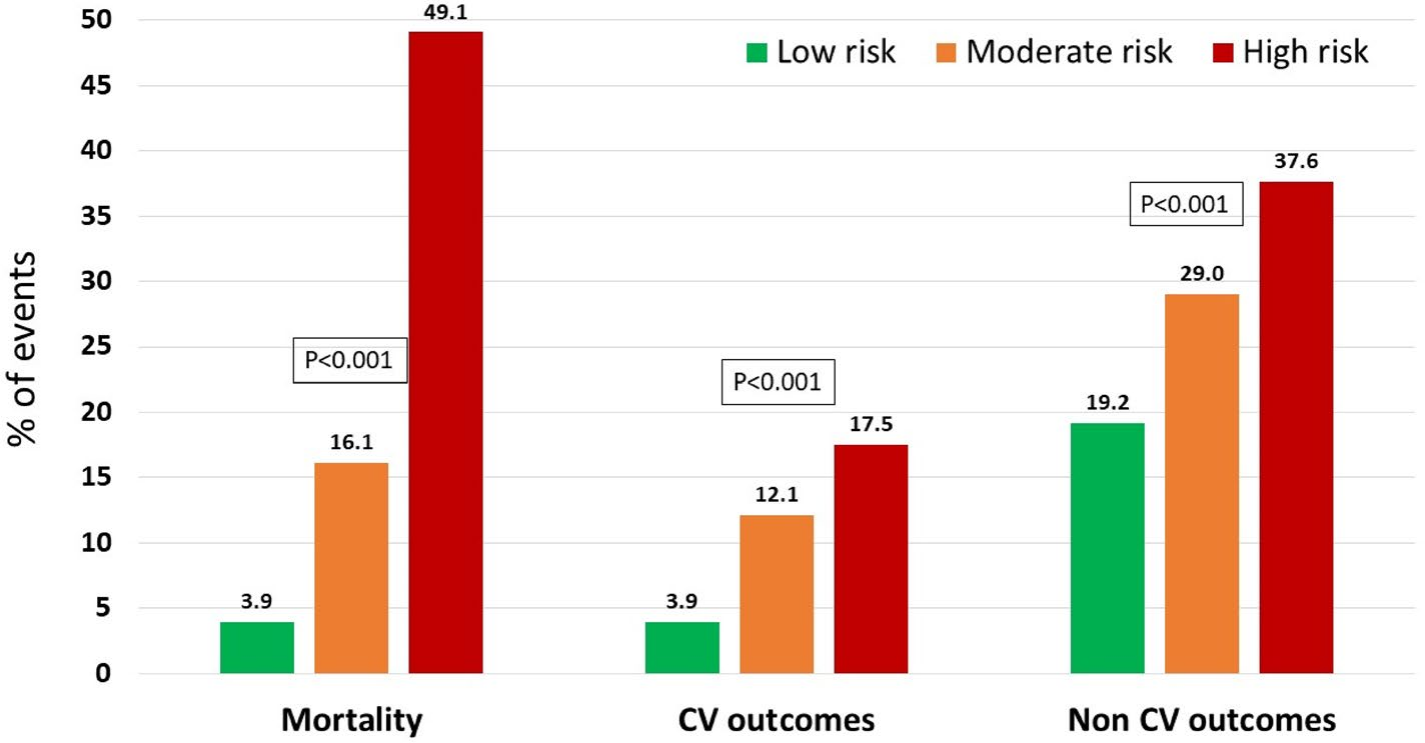
Outcomes by risk groups of COVID-19 lab score. *CV* cardiovascular (myocardial infarction, hospitalizations due to heart failure, stroke).

Depending on the COVID-19 Lab score, mortality varies as shown in Figs. 2 and 3, the higher the score, the poorer the outcome. Three groups were set, dividing patients in low (< 12 points), moderate (12–18 points) and high (19 or higher points) risk of death, with significant mortality differences between them (3.9%, 16.1%, 49.1% respectively, p > 0.001).

The performance of this risk score was tested by assessing its discrimination and calibration capacity for all-cause death. Discrimination was evaluated by calculating the C statistic, 0.85 (0.82–0.88), and calibration was assessed by the Hosmer–Lemeshow test, p-value 0.63 (Table 5).

## Discussion

We constructed a simple score in a cohort of occidental population with COVID-19, which can stratify patients who was admitted to the hospital depending on the risk of death. The most remarkable characteristics of our score are the good performance for mortality risk stratification, that it can be easily obtained. This is because it includes only objective laboratory parameters and due to its dynamic nature, allowing a continuous mortality risk assessment that may help physicians to support clinical decisions in this group of patients. Above all it has to be stated that the experienced clinician and his clinical examination and estimation of the patient´s condition remain indispensable.

On account of this, Covid-19 Lab score could have important implications in the future as COVID-19 disease has very different clinical manifestations depending on the patient, and also depending on the stage of the disease. Because of the inflammation and cytokine storm reported in previous studies, patient´s status can rapidly change from one day to another or even in a shorter period of time^7,10^. Patients with mild or no symptoms can develop acute respiratory insufficiency in a few hours. That is the main reason why not only identifying those cases with higher risk of death, but trying to foresee a clinical deterioration will be mandatory to implement a close monitoring and to use a more aggressive treatment.

Hyperinflammation and cytokine storm are previously described as the most important reasons of bad outcomes in different retrospective studies about SARS-CoV2 infection^5,11^. This uncontrolled immunity response is the main reason why acute respiratory syndrome emerges in this patients, causing clinical deterioration and the need of mechanical respiratory support. Inflammation biomarkers are known to be elevated in COVID-19 disease, such as interleukins (IL), TNFα, ferritin, D-dimer, C-reactive protein (CRP) or procalcitonin^10,12^. Although procalcitonin in COVID-19 is controversially discussed in literature, it has a high correlation with bacterial superinfection, which increases risk of poor outcomes. Akbarshakh et al., showed that some biomarkers like IL-6, ferritin, CRP and troponin were associated with higher mortality. Furthermore, also patients with elevated D-Dimer levels and lymphocytopenia were assessed to have worse outcomes, probably in relation with a procoagulability and immunodeficiency status^3,13,14^.

It is not the first time these types of biomarkers are evaluated in viral infections. Inflammatory reaction and elevated plasma levels were also describe in other viral infections due to other coronavirus like SARS-CoV and MERS-CoV^15–17^. Other study conducted by Bautista et al.^18^ showed that inflammatory biomarkers were associated with higher risk of death in patients with severe acute respiratory syndrome (SARS), secondary to Influenza A H1N1 infection,.

There are also clinical features associated with higher mortality rate in SARS-Cov2 infection. An older age, comorbidities, previous cardiovascular disease, the presence of fever or respiratory insufficiency are associated with poorer outcomes^12–14,19^.

Others scores like CURB-65 and MuLBTSA were previously assessed for predicting mortality in bacterial and viral pneumonias respectively. Although Lingxi et al.^20^ proposed this MuLTBSA score for predicting mortality better than CURB65^21^ in viral pneumonia, it is a static score. This characteristic makes it incapable of showing changes in the risk of death from day to another depending on the progression of the infection and it does not include inflammatory parameters. Furthermore, the main difference with our score is that ours can be replicated very easily only with blood test because all variables are laboratory biomarkers.

Cut-off points for each biomarker were selected depending on the ROC curve in order to achieve the most accurate score. Nevertheless, assuming other cut-off values would change the accuracy of the predicting model.

The clinical manifestations of COVID-19 disease change rapidly in relation with this hyperinflammation reaction, and patients develop acute respiratory insufficiency needing ventilator mechanical support. Having a score like ours, that is not static, it is very simple, and that predicts a hard outcome like mortality will help not only for identifying those patients with high risk of death, but also for anticipating the clinical deterioration. This could be necessary to implement a more aggressive treatment and to keep a close patient monitoring. On the other hand, should be remarked that the experienced clinician and his clinical examination and estimation of the patient´s condition remain indispensable.

Finally, a mobile app that will be available worldwide is being developed to help to calculate quickly the Covid-19 Lab score in order simplify even more the physician’s decisions.

Our study had some limitations. This is a retrospective and observational study, even when this score composed of laboratory biomarkers adjusted by other clinical characteristics emerges to predict mortality in COVID-19 disease in an excellent way, other cofounders might be underestimated. Secondly, antiviral treatment was not the same in all patients, because of the lack of evidence of them at the time of the study. Furthermore, follow up is still ongoing, and some complications or events could be missed. Due to the low sample size, the score was derived and validated in the same cohort with the total study population. Despite these limitations, it is a strong study, including all patients admitted to hospital from three different centres, based on objective parameters. Finally, for a more reliable assessment of our score performance, it must be validated in other cohorts of patients from different centres and geographic areas.

## Conclusion

Covid-19 Lab score is a dynamic simple score only using laboratory biomarkers that can be easily obtained. It can predict in an excellent and dynamic way mortality risk in patients admitted to hospital due to SARS-CoV2 infection in different occasions during the hospital stay. The higher score, the higher mortality risk. Lower hemoglobin o lymphocytes, higher creatitnin levels, higher coagulation and inflammatory biomarkers, were associated with a poorer outcome.

## Supplementary Information

Supplementary Tables.
