# Supplementary material for: The COVID-19 lab score: an accurate dynamic tool to predict in-hospital outcomes in COVID-19 patients
Source: Sci Rep. 2021 Apr 30;11:9361. doi: 10.1038/s41598-021-88679-6 (PMC8087839; doi:10.1038/s41598-021-88679-6)
Supplement: Supplementary file 1 — Supplementary Tables. [file 41598_2021_88679_MOESM1_ESM.docx]

**Supplementary Table 1. Univariate analysis for mortality**

| **Supplementary Table 1. Univariate analysis for mortality** | | | |
| --- | --- | --- | --- |
| **Variables** | **DEATH** | | |
|  | **OR** | **95% CI** | **P** |
| **Demographic characteristics** | | | |
| **Age, per year** | 1.09 | 1.07-1.10 | <0.001 |
| **Female sex** | 1.20 | 0.86-1.68 | 0.288 |
| **Obesity** | 1.13 | 0.69-1.86 | 0.617 |
| **Institutionalized person** | 0.65 | 0.14-2.89 | 0.567 |
| **Dementia** | 0.90 | 0.26-3.18 | 0.874 |
| **Dependency** | 1.30 | 0.63-2.69 | 0.484 |
| **Cardiovascular risk factors** | | | |
| **Active smoking** | 0.59 | 0.23-1.53 | 0.279 |
| **Hypertension** | 2.79 | 1.96-4.00 | <0.001 |
| **Diabetes mellitus** | 2.68 | 1.84-3.90 | <0.001 |
| **Dyslipidemia** | 1.63 | 1.17-2.29 | 0.004 |
| **Cardiovascular disease** | | | |
| **Peripheral artery disease** | 3.29 | 1.36-7.93 | 0.008 |
| **Heart disease** | 2.89 | 1.87-4.45 | <0.001 |
| **Ischemic heart disease** | 2.19 | 1.31-3.68 | 0.003 |
| **Depressed LVEF** | 3.21 | 0.71-14.46 | 0.130 |
| **Valvular heart disease** | 3.08 | 0.96-9.81 | 0.058 |
| **Atrial fibrillation** | 1.84 | 0.70-4.86 | 0.219 |
| **Comorbidities** | | | |
| **Pulmonary disease** | 0.93 | 0.46-1.89 | 0.851 |
| **COPD or Asthma** | 2.19 | 1.34-3.58 | 0.002 |
| **Chronic Kidney Disease** | 1.88 | 1.07-3.30 | 0.029 |
| **Prior stroke** | 0.79 | 0.23-2.74 | 0.707 |
| **Anemia** | 2.68 | 1.89-3.79 | <0.001 |
| **Prior cancer** | 1.42 | 0.38-5.28 | 0.606 |
| **Hypothyroidism** | 0.76 | 0.17-3.48 | 0.729 |
| **Autoimmune disease** | 0.84 | 0.24-2.94 | 0.787 |
| **Prior medical therapy** | | | |
| **Anticoagulation** | 3.52 | 2.33-5.33 | <0.001 |
| **Antiplatelet therapy** | 1.85 | 1.25-2.74 | 0.002 |
| **ACEI/ARB** | 1.73 | 1.23-2.42 | 0.001 |
| **Antialdosteronic drug** | 2.68 | 1.09-6.56 | 0.031 |
| **B-blockers** | 2.85 | 1.94-4.18 | <0.001 |
| **Calcium channel blocker** | 2.16 | 1.25-3.74 | 0.006 |
| **Diuretic drugs** | 3.28 | 2.15-5.01 | <0.001 |
| **Statin** | 1.24 | 0.88-1.76 | 0.226 |
| **Corticosteroid** | 0.38 | 0.09-1.62 | 0.189 |
| **COVID-19 presentation** | | | |
| **Days of symptoms, per day** | 0.89 | 0.86-0.94 | <0.001 |
| **Fever** | 0.41 | 0.29-0.58 | <0.001 |
| **Respiratory insufficiency** | 10.5 | 6.94-15.95 | <0.001 |
| **Medical therapy** | | | |
| **Antiviral** | 0.45 | 0.30-0.68 | <0.001 |
| **Chloroquine** | 0.64 | 0.41-1.01 | 0.053 |
| **Interferon** | 1.37 | 0.61-3.09 | 0.450 |
| **Tocilizumab** | 0.82 | 0.46-1.46 | 0.496 |
| **Azithromycin** | 1.17 | 0.81-1.69 | 0.410 |
| **Ceftriaxone** | 1.91 | 1.35-2.68 | <0.001 |
| **Corticosteroids** | 2.12 | 1.48-3.02 | <0.001 |
| **Anticoagulation** | 1.89 | 1.35-2.65 | <0.001 |
| **Antiplatelets** | 1.77 | 1.23-2.53 | 0.002 |

**Supplementary Table 2. Events according to COVID-19 Lab Score**

| **Supplementary Table 2. Events according to COVID-19 Lab Score** | | | |
| --- | --- | --- | --- |
| **Score** | **Rates of outcomes (%)** | | |
|  | **Mortality** | **CV outcomes** | **Non-CV outcomes** |
| 0 | 0 | 0 | 19.4 |
| 1 | 0 | 12.5 | 25.0 |
| 2 | 0 | 0 | 16.7 |
| 3 | 0 | 12.5 | 25.0 |
| 4 | 1.5 | 1.5 | 16.9 |
| 5 | 0 | 4.2 | 8.3 |
| 6 | 2.4 | 0 | 2.4 |
| 7 | 5.1 | 2.6 | 20.5 |
| 8 | 1.7 | 1.7 | 31.0 |
| 9 | 4.8 | 7.1 | 11.9 |
| 10 | 7.8 | 7.8 | 33.3 |
| 11 | 15.2 | 9.1 | 15.2 |
| 12 | 3.1 | 12.5 | 18.8 |
| 13 | 9.3 | 9.3 | 30.2 |
| 14 | 13.6 | 4.5 | 18.2 |
| 15 | 22.9 | 17.1 | 54.3 |
| 16 | 14.6 | 12.2 | 22.0 |
| 17 | 27.8 | 19.4 | 33.3 |
| 18 | 29.4 | 11.8 | 29.4 |
| 19 | 25.6 | 12.8 | 33.3 |
| 20 | 37.5 | 20.8 | 41.7 |
| 21 | 33.3 | 14.3 | 38.1 |
| 22 | 40.0 | 20.0 | 45.0 |
| 23 | 35.7 | 21.4 | 28.6 |
| 24 | 52.4 | 19.0 | 23.8 |
| 25 | 65.0 | 15.0 | 40.0 |
| 26 | 44.4 | 16.7 | 33.3 |
| 27 | 76.5 | 29.4 | 35.3 |
| 28 | 100 | 12.5 | 50.0 |
| 29 | 75.0 | 25.0 | 0 |
| 30 | 75.0 | 15.0 | 55.0 |
